# Factors Affecting Immune Reconstitution Post‐Allogeneic HSCT in Children: The Case for an Individualized Approach to Vaccination

**DOI:** 10.1111/ejh.70085

**Published:** 2025-12-18

**Authors:** Hélène Buvelot, Frederic Baleydier, Laure Pittet, Geraldine Blanchard‐Rohner

**Affiliations:** ^1^ Centre for Vaccinology Geneva University Hospital Geneva Switzerland; ^2^ Division of Pediatric Oncology and Hematology, Department of Women, Child, and Adolescent Geneva University Hospitals Geneva Switzerland; ^3^ Cansearch Research Platform for Pediatric Oncology and Hematology, Department of Pediatrics, Gynecology, and Obstetrics, Faculty of Medicine University of Geneva Geneva Switzerland; ^4^ Unit of Immunology, Vaccinology, and Rheumatology, Division of General Pediatrics, Department of Pediatrics, Gynecology and Obstetrics, Geneva University Hospitals University of Geneva Geneva Switzerland

**Keywords:** HSCT, immune reconstitution, vaccination, vaccine response

## Abstract

Allogeneic hematopoietic stem cell transplantation (HSCT) is increasingly used to treat malignant and non‐malignant diseases. Following allogeneic HSCT, patients are particularly vulnerable to vaccine‐preventable diseases (VPD) because conditioning depletes immune cells, including memory cells. Revaccination is therefore essential, but multiple factors, such as conditioning regimen, stem cell source, HLA compatibility, graft‐versus‐host‐disease (GVHD), and age, affect immune reconstitution and vaccine response. Current guidelines recommend uniform vaccination schedule for all allogeneic HSCT patients, despite this heterogeneity. In this review, we discuss how these factors influence immune reconstitution and vaccine response, and highlights the need for a more individualized approach. Based on current evidence, we propose that vaccine timing, particularly for inactivated vaccines, could benefit from adjustment according to immune recovery markers, such as lymphocyte counts and presence of GVHD, rather than relying on fixed post‐HSCT timepoints. We also discuss emerging immunotherapies, including CAR‐T cells and bispecific antibodies, which can induce similarly prolonged immunosuppression and may benefit from personalized vaccination strategies. Further studies in pediatric populations are needed to define immunological threshold that would enable safer and more effective personalized vaccination schedules.

## 1 Introduction

Hematopoietic stem cell transplantation (HSCT) is a cellular therapy increasingly used to treat various diseases, including malignant hematologic diseases (e.g., leukemia), non‐malignant hematologic diseases (e.g., sickle cell disease), inborn errors of metabolism (e.g., lysosomal storage disease), and primary immunodeficiencies (e.g., severe combined immunodeficiency). Before transplantation, conditioning is done using chemotherapy with or without radiotherapy, depending on the underlying disease, the source of stem cells used, as well as the degree of human leukocyte antigen (HLA) compatibility between the donor and the recipient [1].

**FIGURE 1:**
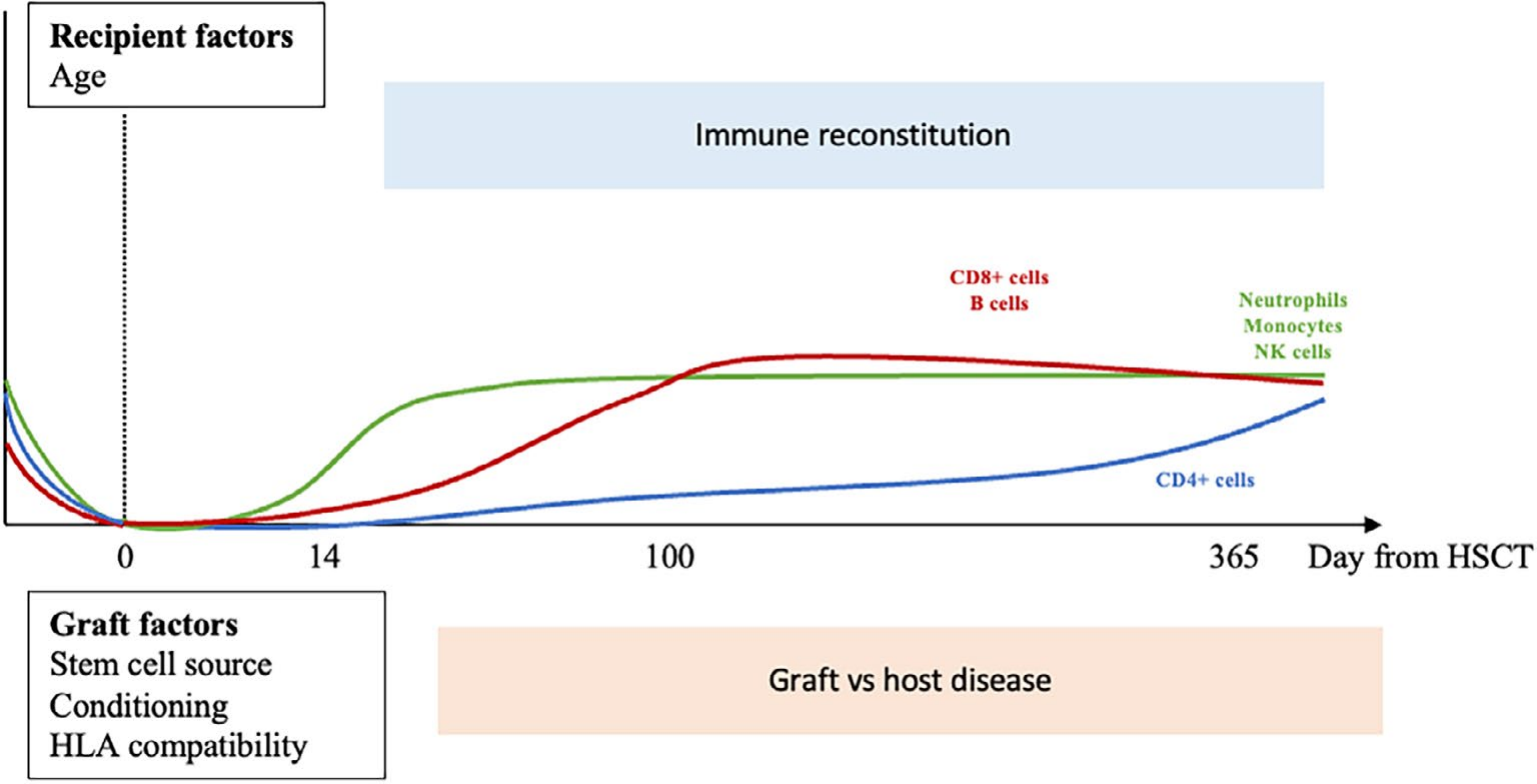
Factors impacting immune reconstitution post‐HSCT. Innate immunity is the first to recover (green line), in the first weeks after transplantation. This is followed by the recovery of B cells and CD8+ T cells (red line). The recovery of CD4+ T cells is the last to occur and can take years. Recipient and graft factors can both delay immune system recovery (adapted from [1]).

Following transplantation, the immune system gradually recovers but it can take several years until being fully functional (Figure 1) [2]. Transplanted patients are hence at higher risk of infections during this period [3]. In addition to the delay in immune reconstitution, other factors contribute to the patient's immunosuppression, such as immunosuppressive treatments and the occurrence and treatment of graft‐versus‐host‐disease (GVHD). Infectious diseases are therefore among the most common complications and a main cause of mortality and morbidity in this population [4, 5]. This is why various prophylactic measures are recommended, including vaccinations. As vaccine‐induced antibody titers decline rapidly after allogeneic HSCT [6], revaccination is a crucial strategy to improve survival [7], and immune recovery‐based vaccination reviews highlight the need for tailored strategies considering immune recovery, GVHD status, and CAR‐T therapy context [8, 9].

Recent mRNA vaccine technology has shown strong immunogenicity and rapid adaptability against emerging pathogen, such as SARS‐CoV‐2. Although pediatric HSCT data are limited, early evidence suggest that mRNA vaccines are safe and can elicit measurable humoral and cellular responses [10], even in immunocompromised patients, underscoring the potential value of incorporating these vaccines into post‐HSCT immunization strategies.

Current guidelines from the European Conference on Infections in Leukemia (ECIL) [11] and from the Infectious Diseases Society of America (IDSA) [12] recommend vaccinations at fixed time‐points beginning 3–6 months after HSCT, for all recipients regardless of differences in conditioning, stem cell source, HLA compatibility, GVHD and age (Table 1).

**TABLE 1:** Recommended vaccination schedule in children after hematopoietic stem cell transplantation from ECIL and IDSA [11, 12].

Vaccine	Recommendations	Comments
Pneumococcal vaccine	3 doses of highest‐valent pneumococcal conjugate vaccine available locally (e.g., PCV15 or PCV20) at 1‐month intervals from 3 months after transplantation	No age adaptation
Diphtheria‐tetanus acellular pertussis‐inactivated polio‐ *Haemophilus influenzae* type b (Hib)‐Hepatitis B vaccine (HBV)	3 doses of combined vaccine at 1–2 months intervals from 6 months after transplantation	< 5 years: DTaP‐IPV‐HBV‐Hib ≥ 5 years: DTaP‐IPV ± HBV (Hib not routinely indicated)
Meningococcal vaccination (ACWY and B):	2 doses of MenACWY conjugate vaccine and 2 doses of a MenB vaccine, given at 2‐month intervals, beginning 6 months post‐transplantation	According to national age‐ and risk‐based recommendations
HPV vaccine	2 or 3 doses from 6 months after transplantation	According to age and country recommendations, but note that the 3‐dose series is preferred for immunocompromised patients
Live attenuated MMR vaccine	2 doses at 1‐month interval from 24 months after transplantation only in seronegative patients and if absence of contra‐indication	Patients should have no ongoing immunosuppression, no GVHD, no recent immunoglobulin treatment in the previous 3 months (ideally in the previous 8 and 11 months, as earlier vaccination may be affected by residual passive immunity), no relapse of the underlying disease
Live attenuated varicella vaccine	2 doses at 1‐month interval from 24 months after transplantation only in seronegative patients and absence of contra‐indications	Patients should have no ongoing immunosuppression, no GVHD, no recent immunoglobulin treatment in the previous 3 months (ideally in the previous 8 and 11 months), no relapse of the underlying disease
Inactivated influenza vaccine	From 3 months after transplantation, 1 dose of seasonal inactivated influenza vaccine during flu season	Children aged 6 months to 8 years receiving influenza vaccination for the first time should receive two doses administered 1 month apart
SARS‐CoV‐2 vaccine	≥ 3 months post‐HSCT; 3‐dose primary series (interval ≥ 3 weeks between doses 1 and 2 and preferably ~3 months between doses 2 and 3)	Annual boosters while immunocompromised (adjust per national guidance)

However, great discrepancies in vaccine responses have been observed among allogeneic HSCT recipients, reflecting differences in immune recovery [13, 14]. Indeed, immunophenotypic analysis in pediatric HSCT recipients have identified specific B‐ and T‐cell subsets (naïve B, naïve CD4^+^) that predict influenza vaccine immunogenicity [15]. In addition, studies in pediatric and young adult HSCT recipients revealed suboptimal protective antibody titers against diphtheria, tetanus and pertussis long after transplantation [16].

In this narrative review, we examine the data available on vaccine responses after allogeneic HSCT, and its influencing factors and discuss the need for individualized vaccination strategies that consider immune recovery markers to optimize protection. Relevant prospective studies on vaccine response after HSCT are summarized in Table 2, and studies on possible influencing factors on the immune reconstitution are summarized in Table 3.

**TABLE 2:** Vaccine response after stem cell transplantation.

Citation, date, country, study design population size	Objectives	Vaccine	Underlying disease, graft source	Study group, median age	Median time to vaccination initiation	Immunogenicity	Key results	Strengths and weakness
Matkowska‐Kocjan et al. [10], Poland	To evaluate the safety and efficacy of the mRNA SARS‐CoV‐2 vaccine	mRNA	Severe aplastic anemia (10)	Children with allo‐HSCT	36 months (range from 5 to 120 months)	Number of patients with positive SARS‐CoV‐2 IgG before vaccination	All reported adverse events were classified as mild	S: prospective design, safety data
Prospective observational cohort study		BNT162b2 (Pfizer/BioNTech) vaccine	ALL (6)	Median age 7 years		– HSCT group: 23/34	HSCT recipients with undergoing immunosuppressive therapy had poorer immunological response than recipients off treatment	
			JMML (4)			– Controls: 25/35		
			AML (2)			Number of patients with anti‐SARS‐CoV‐2 IgG after the 2nd dose of vaccine		
			Wiskott‐Aldrich syndrome (2)			– HSCT group: 34/34		
			Lymphoma (2)	Controls		– Controls: 35/35	Age, time since transplant, lymphocyte count, and total IgG concentration did not correlate with anti‐SARS‐CoV‐2 concentration	W: non‐randomized design, small sample size, antibody‐only analysis, short follow‐up
			CML (1)			Anti‐SARS‐CoV‐2 IgG concentration after 2 doses of vaccine		
34 pediatric allogeneic HSCT recipients and 35 healthy controls			Hemophagocytic syndrome (1)	Median age 7 years		– HSCT group: 0.1–1448 BAU/mL		
			SCID (1)					
			Adrenoleukodystrophy (1)			– Controls: 1481–5680 BAU/mL		
			Diamond‐Blackfan anemia (1)					
			X‐linked agammaglobulinemia (1)					
			Mucopolysaccharidosis (1)					
Amarin et al. [15], USA	To identify B‐ and T‐cell subpopulations associated with influenza vaccine immunogenicity	High‐dose trivalent influenza vaccine and standard‐dose quadrivalent influenza vaccine	ALL (39)	Children with allo‐HSCT	8 months (range from 4.6–13.6 months)	Significant association between high absolute CD4+ and CD19+ numbers at baseline and increased vaccine immunogenicity	Identification of 7 distinct immune B‐ and T‐cell subpopulations associated with vaccine response	S: High‐dimensional immune profiling, large sample size, sensitivity analyses
			AML (29)					
			CML (3)					
			MDS (5)					
Multicenter, double‐blinded, randomized controlled trial			Other malignancy (8)					
			Severe aplastic anemia (25)	Median age 11.8 years		No evidence for an association between the absolute CD8+ number at baseline and vaccine immunogenicity, except for A/H3N2		W: cross‐sectional study design, not adjusted for immunosuppressive therapy or GVHD
			Inherited erythrocyte abnormalities (30)					
156 pediatric allogeneic HSCT recipients			Immune system disorder (6)					
			Fanconi anemia (6)					
			Other non‐malignant (5)					
Inaba et al. [17], USA	To assess long‐term vaccine‐induced antibody responses in pediatric HSCT recipients and evaluate the persistence of immunity over time	Diphtheria, tetanus, pertussis, MMR, HBV and IPV	AML (61)	Children with allo‐HSCT	– Vaccination series began ≥ 12 months post‐transplant (per institutional schedule: DTaP, IPV, HBV: 12, 15, 18 months, MMR: 24 months).	Antibody responses lasting more than 5 years after immunization	Most children regain protective titers post‐revaccination; chronic GVHD reduces responses	S: Long‐term follow‐up, large pediatric cohort, comprehensive serologic data
			ALL (48)					
			CML (28)		Duration of follow‐up: Up to 5 years after HSCT.			
			Aplastic anemia (18)			– Tetanus (95.7%)		
Prospective longitudinal observational study			MDS (17)	Median age 8.5 years		– Rubella (92.3%)	Factors associated with vaccine failure were older age at immunization, low CD3+, CD4+ or CD19+, high IgM concentrations, positive recipient CMV serology, negative titers before immunization, acute or chronic GVHD, radiation during preconditioning	W: Single‐center, antibody‐only analysis, heterogeneous population
			Sickle cell (14)			– Poliovirus (97.9%)		
			Immune disorder (14)			– Pertussis (25%)		
116 pediatric allogeneic HSCT recipients.			Others (10)			– Measles (66.7%)		
			BM (172)			– Mumps (61.5%)		
			PBSC (38)			– HBV (72.9%)		
Cordonnier et al. [18], Infectious Disease Working Party of the European Group for Blood and Marrow Transplantation	To compare immunogenicity and safety of early versus late initiation of pneumococcal conjugate vaccination after allogeneic HSCT	PCV7	Acute leukemia (88)	Adults with allo‐HSCT	Early vaccination group: 3 months	Response rate in early vaccination group: 45/57 (79%)	Early vaccination safe and immunogenic; comparable to late vaccination	S: RCT design, multicenter, included children, strong methodological rigor
			Chronic leukemia (36)					
			Lymphoma (8)					
			Plasma cell disorders (1)					
			MDS or myeloproliferative syndrome (11)					
Multicenter, open‐label, randomized controlled trial (RCT).			Aplastic anemia (12)	Median age: 37 years	Late vaccination group: 9 months	Response rate in late vaccination group: 47/57 (82%)		W: Mixed ages, short follow‐up, PCV7 now outdated, immunogenicity only
			Hemoglobinopathy (2)					
			BM (83)					
158 allogeneic HSCT recipients (adults and children).			PBSC (73)			Response rate was defined as antibody level ≥ 0.15 𝜇g/ml for each of the 7 serotypes in the vaccine		
			BM and PBSC (1)					
			CB (1)					
Mohty et al. [19], Switzerland	To evaluate the immunogenicity and determinants of response to the AS03‐adjuvanted monovalent influenza A(H1N1) pdm09 vaccine in allogeneic HSCT recipients	AS03‐adjuvanted influenza A/09/H1N1	AML (20)	Adults with allo‐HSCT	25 months (range 3–220 months)	After 2 doses, HSCT recipients achieved a seroprotection rate in 84% of the patients	Seroprotection 47% after one dose, 72% after two; Most significant predictors of poor antibody response = short transplant‐to‐vaccination interval (< 12 months) and active GvHD	S: Controlled design, strong clinical data, mechanistic insight on GVHD
			Lymphoma (12)					
			CML (9)					
Prospective observational study.			ALL (5)	Median age 52 years				
			MDS (5)					
			Aplastic anemia (4)					
82 allogeneic HSCT recipients (adults and adolescents ≥ 12 years).			Multiple myeloma (4)					W: Mostly adults, one‐season data, antibody‐only, no long‐term follow‐up
			CLL (3)					
			Myeloproliferative syndrome (3)					
Cordonnier et al. [20], Infectious Disease Working Party of the European Group for Blood and Marrow Transplantation	To assess the functional quality of anti‐pneumococcal antibodies by comparing IgG concentrations and OPA after immunization with PCV7 in allogeneic HSCT recipients	PCV7	Acute leukemia (9)	Adults with allo‐HSCT	Early vaccination group: 3 months	Correlation between pneumococcal IgG ELISA titers and OPA titers against each of the antigens included in PCV‐7	IgG titers strongly correlated with opsonophagocytic activity; no difference between early/late schedules	S: Functional OPA data, RCT‐linked cohort, validated assays
Laboratory‐based substudy (immunologic analysis) linked to the 2009 multicenter RCT			Chronic leukemia (13)			– pn4: *r* = 0.76		
57 allogeneic HSCT recipients drawn from the original RCT cohort.						– pn6b: *r* = 0.71		
			MDS (3)			– pn9v: *r* = 0.70		
			Aplastic anemia (2)	Mean age: 37 years	Late vaccination group: 9 months	– pn14: *r* = 0.48		W: Small sample, short‐term follow‐up, mixed ages, PCV7 outdated
			Lymphoma (1)			– pn18c: *r* = 0.70		
			BM (14)			– pn19f: *r* = 0.74		
			PBSC (14)			– pn23f: *r* = 0.75		
Pao et al. [21], USA	To evaluate antibody responses to PCV7 and Hib conjugate vaccines in pediatric and adult allo‐HCT recipients, and to examine factors affecting immunogenicity, including age, GVHD, and immunosuppression	PCV‐7, Hib	Acute leukemia (64)	Adults and children with allo‐HSCT	6–12 months post‐transplant depending on immune recovery	PCV‐7 response	Pediatric patients: 80%–90% achieved protective titers; adults lower; GVHD and immunosuppression reduced responses	S: Pediatric/adult comparison, prospective, safety data, identification of predictors
			Chronic leukemia (6)					
						– Adults 34/76 (44%)		
						– Children 45/51 (88%)		
						Hib response		
						– Adults: 51/65 (79%)		
Prospective cohort study			Aplastic anemia/MDS (26)	Median age: 23 years		– Children: 48/50 (96%)		W: Moderate size, short‐term only, IgG titers only, single center
77 allo‐HCT recipients (42 pediatric [< 18 years] and 35 adults).			NHL/HD (13)			PCV‐7 and Hib vaccine response was defined as seroconversion or > 3‐fold rise in titers.		
			Others (18)			PCV‐7 response in ≧ 50 years was better when CD4+ > 200/μL, IgG > 500 mg/dL and PHA response within 60% lower limit of normal		
Karras et al. [22], USA	To compare immunogenicity of one versus two doses of seasonal trivalent inactivated influenza vaccine in pediatric and adult allogeneic HSCT recipients	Influenza trivalent vaccine: 1 vs. 2 doses	ND	Adults and children with allo‐HSCT	≥ 6 months post‐HSCT, during the influenza season	No statistical differences in the rates of seroprotection and seroconversion in patients who received 1 or 2 doses of vaccine	Immune reconstitution and interval from transplant were stronger determinants of vaccine response than the number of doses beyond the first	S: RCT design, pediatric inclusion, multicenter, standardized serology, safety monitored
Prospective, multicenter randomized trial.				Median age: 40 years		Seroprotection was defined as vaccine titer > 1:40 at 8 weeks post‐vaccination		W: Small pediatric sample, short‐term follow‐up, one season only, antibody‐only measurement
82 allogeneic HSCT recipients (26 pediatric [< 18 years] and 56 adults).						Seroconversion was defined as > 4 fold increase in Ab titer at 8 weeks post‐vaccination		

Abbreviations: aGVHD, acute graft vs. host disease; ALL, acute lymphoid leukemia; AML, acute myeloid leukemia; ATG, anti‐thymocyte globulin; BAU, binding antibody units; BM, bone marrow; CB, cord blood, cGVHD, chronic graft vs. host disease; CML, chronic myeloid leukemia; HBV, hepatitis B virus; HD, Hodgkin disease; HI, hemagglutinin inhibition assay; Hib, *Haemophilus influenzae* type B; HSCT, hematopoietic stem cell transplantation; IPV, inactivated poliovirus; MDS, myelodysplastic syndrome; MMR, measles, mumps and rubeola; ND, not determined; NHL, non‐Hodgkin lymphoma; OPA, opsonophagocytic activity; PBSC, peripheral blood stem cells; PCV, pneumococcal conjugated vaccine; Tdap, tetanus, diphtheria and acellular pertussis.

**TABLE 3:** Study of factors influencing immune reconstitution after HSCT.

Citation, date, country, study design, population size	Objectives	Participants (*n*)	Underlying disease	Study group	Outcome	Follow up	Key results	Strengths and weakness
Charrier et al. [23], Canada.	To characterize lymphocyte reconstitution, focusing on maturing and regulatory T and B cell subsets, in pediatric recipients of cord blood (CB) and bone marrow (BM) transplants	40 children (median age 10.8 years)	Malignant (30)	BM (12)	Immune reconstitution after CB and BM transplant	12 months	Early regulatory T‐cell recovery, delayed naive T‐ and memory B‐cell recovery; CB slightly slower naive T‐cell reconstitution	S: Pediatric cohort, longitudinal design, detailed immunophenotyping, CB vs. BM comparison
Prospective observational study			– ALL (14)		– B cell count			
			– AML (7)		– CD4+ cell count		B cell count was higher at 3 months post‐transplant with CB than BM	
			– CML (2)	CB (28)	– CD8+ cell count		No difference in CD4+ and CD8^+^ cell count recovery that remain low during the first 9 months post‐transplant	W: Small sample, single center, 1‐year follow‐up, no functional assays, no direct clinical outcome correlation
41 children (21 CB, 20 BM)			– MDS (6)		– NK cell count		NK cells were higher after CB transplant at 3, 9 and 12 months post‐transplant	
			Non‐malignant (10)					
Rao et al. [24], UK.	To evaluate safety, engraftment, immune reconstitution, and survival after reduced‐intensity conditioning (RIC) in children with primary immunodeficiency (PID) undergoing unrelated donor bone marrow transplantation (BMT)	52 children (median age RIC 5, 9 years, median age MAT 1, 9 years)	SCIDs (13)	RIC (33)	Events after HSCT	Median follow‐up in RIC group: 40 months	No statistical differences in the speed of immune reconstitution or graft‐vs‐host disease	S: Pediatric focus, comprehensive immune monitoring
			Non‐SCIDs (39)		– GVHD			
Prospective single‐arm interventional cohort	RIC regimen: fludarabine, melphalan, and alemtuzumab or anti–thymocyte globulin		– WAS (5)		– Viral reactivations		More viral reactivations in the RIC group (39%) than in the MAC group (21%)	
			– T‐cell deficiency (23)	MAC (19)	– Mortality	Median follow up in MAC group: 104 months		W: Small, single center, heterogeneous diseases, short follow‐up, no control arm
			– CD40 ligand deficiency (6)		Compared to a retrospective cohort with MAC		Overall survival better in the RIC group (94%) compared with MAC group (47%)	
52 children	MAC regimen: bisulphan and cyclophosphamide		– Phagocytic disorders (5)					
Olkinuora et al. [25], Finland.	To characterize immune reconstitution in pediatric HSCT recipients and to determine how early viral infections and graft‐versus‐host disease (GVHD) influence T‐ and B‐cell recovery and function	51 children (mean age 8 years)	Malignant (39)	Children with HSCT	Impact of early (< 100 days) viral reactivation/infection and GVHD on immune recovery	18 months	Early viral infections delay CD4^+^ T‐cell recovery; GVHD impairs B‐cell reconstitution; NK cells recover fastest	S: Pediatric‐only cohort, detailed immune profiling, clinical correlations (GVHD, viral infections)
Prospective observational cohort study			Non‐malignant (12)				Early viral infections and prolonged T cell immunodeficiency and thymic dysfunction may be indicative of subclinical GVHD	W: Moderate sample, heterogeneous cohort, no functional assays, short follow‐up, single center
44 children undergoing allogeneic HSCT								

Abbreviations: aGVHD, acute graft vs. host disease; ALL, acute lymphocytic leukemia; AML, acute myeloid leukemia; BM, bone marrow; CB, cord blood; cGVHG, chronic graft vs. host disease; CML, chronic myeloid leukemia; HLH, hemophagocytic lymphohistiocytosis; HSCT, hematopoietic stem cell transplantation; JMML, juvenile myelomonocytic leukemia; MAC, myeloablative conditioning; MDS, myelodysplastic syndrome; ND, not determined; NHL, non‐Hodgkin's lymphoma; PBSC, peripheral blood stem cells; RIC, reduced intensity conditioning; SAA, severe aplastic anemia; TBI, total body irradiation; WAS, Wiskott‐Aldrich syndrome.

## 2 Stem Cell Sources

Allogeneic stem cells used for HSCT can be sourced either directly from the donor's bone marrow (BM), from peripheral blood stem cells (PBSC) or from cord blood (CB) [26]. The differences in graft composition is known to impact the immune reconstitution. However, limited data is available for pediatric populations [27]. Unfortunately, findings derived from adult studies cannot be directly transposed to children, as there are key differences between these two populations, including a higher rate of GVHD in adults [28]. The few studies that have focused on pediatric patients have shown that CB recipients have a faster recovery of naïve and memory B cells at 6 months after HSCT, but a slower T cell recovery compared to BM and PBSC recipients [23, 29, 30]. PBSC transplant recipients have a faster lymphocyte recovery compared to BM but a subsequent increased risk of chronic GVHD, according to studies involving both pediatric and adult patients [30, 31, 32]. These differences in immune recovery could theoretically influence vaccine responses post‐HSCT, but this has not been thoroughly investigated yet [33]. One study reported that the response to protein‐conjugated vaccines was similar between CB and non‐CB recipients in both adult and pediatric patients [34]. Current vaccination guidelines do not differentiate between stem cell sources used for HSCT [11, 12]. Further studies are needed to assess the influence of stem cell sources on vaccine immune responses and whether vaccination guideline should be adapted accordingly.

## 3 Conditioning

Conditioning is a necessary step before HSCT to ensure adequate transplant engraftment, immunosuppression and destruction of residual cancer cells in case of malignant diseases [1]. Myeloablative conditioning (MAC) regimens are harsher on the immune system compared to reduced toxicity conditioning (RTC), reduced intensity conditioning (RIC) and non‐myeloablative (NMA) regimens [35]. It has been hypothesized that the reduced thymic toxicity and the residual host cells that have survived conditioning in RIC might lead to a better and faster immune reconstitution following HSCT [36]. The few studies comparing immune reconstitution and vaccine responses in RIC versus MAC pediatric HSCT recipients have shown mixed results, with one study showing a better immune reconstitution in RIC regimens [37], while some showed no difference [24, 38]. However, RIC regimens are mainly prescribed in older patients, as children have a better tolerance to myeloablative conditioning. Therefore, studies comparing vaccine responses between conditioning regimens are difficult to perform because in pediatric population [1]. As RTC regimens are relatively new, only few studies have studied vaccine responses with such conditioning [1]. Current guidelines do not take into account conditioning regimens in their vaccination schedule [12].

## 4 HLA Compatibility

HSCT donors can either be HLA matched, HLA mismatched or HLA haploidentical. Maximal HLA compatibility between donor and recipient is of utmost importance in HSCT, as HLA disparities have been linked with an increased risk of GVHD, delayed immune reconstitution, as well as graft failure and mortality [39, 40, 41]. To mitigate the increased risk of GVHD, modifications are done to the HSCT process in the case of HLA mismatch. Either the pre‐transplantation conditioning regimen and post‐transplantation immunosuppression are intensified, or the graft is manipulated with donor T cell depletion [42]. This T cell depletion, while efficient in decreasing the incidence of GVHD and diminishing the need for post‐transplantation immunosuppression, contributes to the delayed immune reconstitution seen in these patients, which negatively impacts vaccine responses [43]. Studies evaluating vaccine responses in HLA mismatched HSCT patients and comparing them to HLA matched are scarce, particularly in the pediatric population [39, 44, 45]. Currently, the vaccination schedule remains the same regardless of the HLA compatibility between donor and recipient [12].

## 5 Recipient Age

Both T cell and B cell must be quantitatively and qualitatively restored to ensure a proper vaccine response. After HSCT, the T cell pool reconstitution occurs through two main pathways. Early after HSCT, T cell clones rapidly expand peripherally, but their diversity is poor. Later on, thymic recovery allows for the development of a diversified T‐cell receptor range [44, 46]. Studies in children have shown that older age at transplantation (i.e., adolescence) is negatively correlated with T cell lymphopoiesis and function [17]. However, in patients aged less than 16 years, T cell lymphopoiesis was primarily influenced by chronic GVHD [47]. The influence of age on T cell lymphopoiesis has been attributed to the natural decline in thymic function with age [48, 49]. Studies focusing on pediatric populations are lacking. Currently, guidelines recommend using the same schedule for all ages, despite evidence that younger patients respond better to some vaccines than older patients [12].

## 6 Graft Versus Host Disease

GVHD is a major complication of HSCT, which occurs when donor cells recognize the recipient's antigens as non‐self and attack host tissues, particularly the skin, gastrointestinal system and liver. Both acute and chronic GVHD impair the recovery of lymphocyte subsets necessary for effective vaccine responses. Studies in adult and children demonstrate lower B cells counts in patients with GVHD, primarly due to bone marrow destruction and cytokine storm associated with GVHD [50, 51, 52, 53]. Chronic GVHD also impairs antibody quality via defective class switching and affinity maturation [50, 51, 54]. T cell count and function are similarly reduced due to thymic damage [47, 55]. This deficiency in lymphopoiesis is exacerbated by GVHD therapies (usually corticosteroids) [54, 56]. Vaccine responses are variably affected [52, 54]: response to conjugated *Haemophilus influenzae* type B (Hib) and pneumococcus (PCV) vaccines are relatively preserved [18, 57, 58, 59], whereas influenza vaccine responses are severely impaired [19, 53].

In a single‐center pilot study evaluating BNT162b2 mRNA COVID‐19 vaccine in 31 adult allogeneic HSCT recipients, 74% developed detectable anti‐SARS‐CoV‐2 antibodies [60]. Chronic GVHD was strongly associated with poor vaccine responses. Active GVHD remains a contraindication for live‐attenuated vaccines, but non‐live vaccines can generally be administered. Current guidelines recommend starting immunization with non‐live vaccines, especially pneumococcal conjugate vaccines, as early as 3 months after HSCT, regardless of the presence of GVHD [11, 12]. Indeed, HSCT patients with GVHD are particularly vulnerable to invasive pneumococcal disease [61]. However, if vaccines are administered during active GVHD, antibody responses should be measured before and after vaccination, as additional doses of vaccines might be necessary to fully ensure protection [62]. Although GVHD does not warrant postponing vaccinations with non‐live vaccines, patients must be GVHD free to receive live‐attenuated vaccines given the theoretical risk that an attenuated strain can revert to the pathogenic form and cause severe infections [62].

## 7 Immune Reconstitution

The reconstitution of a fully functional immune system takes years (Figure 1). It begins with the relatively rapid recovery of innate immune cells, represented by neutrophils and NK cells, followed by T cells. CD4^+^ T cells, which are particularly important for subsequent response to vaccination, emerge several months after CD8^+^ T cells. B cell recovery, both in number and function, is particularly delayed in unconditioned transplants or after reduced intensity conditioning is used [63, 64]. During this period, patients are highly susceptible to common and opportunistic infections, especially *S. pneumoniae* , influenza and Hib [59, 65, 66]. Active immunization, together with early recognition and treatment of infections, are therefore essential during this period. The optimal timing to initiate immunization after HSCT is unknown and may depend on the degree of immune reconstitution, intensity of immunosuppressive therapy, national epidemiology, as well as individual patient characteristics [25, 67]. Recent data from adult allogeneic HSCT recipients demonstrated that the interval between transplantation and vaccination strongly influences vaccine immunogenicity. In a JAMA Network Open cohort, higher anti‐pre‐F RSV IgG titers were observed in adult HSCT recipients vaccinated later post‐transplant and in those with higher lymphocyte counts at the time of vaccination [68]. Similarly, a JAMA research letter reported attenuated immune responses among adult patients vaccinated earlier post‐transplant, highlighting the impact of incomplete immune recovery on vaccine responsiveness [69]. In line with these findings, Haynes et al. demonstrated that pediatric HSCT patients who had individualized vaccine timing based on lymphocyte recovery achieved improved serological responses [70]. It is important to note that current RSV vaccines are not approved for pediatric use, meaning that post‐HSCT vaccination strategies involving RSV immunization are presently restricted to adults. In a prospective observational study, adult HSCT recipients were immunized against SARS‐CoV‐2 using one of the two available mRNA vaccines following transplantation. The overall serological response rate was 79% in patients vaccinated within the first year post‐HSCT, 89% between 1 and 3 years and 92% in those vaccinated more than 3 years after transplant [71]. Immune reconstitution parameters, particularly CD4^+^, CD19^+^ and IgG levels, were strongly associated with the likelihood of developing a serological response. Immunosuppressive therapies, such as ruxolitinib, cyclosporine or tacrolimus used for GVHD treatment or prophylaxis, did not significantly reduce the probability of developing spike‐specific antibodies, although recipients on these agents were less likely to mount neutralizing antibodies. In contrast, oral steroid use was associated with reduced spike antibody titers and diminished neutralizing activity.

At a cellular level, effective T‐ and B‐cells interactions are required to achieved robust and long‐lasting vaccine immunity. This explains why low T cell and/or B cell counts are associated with weaker vaccine responses [18, 20, 21, 72]. Different cell‐count predictors have been reported, with absolute CD19^+^ cell counts being a predictor of influenza, Hib, tetanus, poliomyelitis, pertussis and diphtheria vaccine responses [17, 22, 34] and CD3^+^ counts being a predictor of antibody responses to measles vaccine [17], while others have observed no effect [6, 7]. Further research is needed to determine which cell types and threshold can reliably predict vaccine responses [73]. However, cell counts alone might not be sufficient to predict vaccine response, as cells can be functionally deficient [56].

mRNA vaccines require coordinated T‐ and B‐cell interactions to generate optimal humoral and cellular responses [74]. Consistent with this, studies in adults post‐HSCT have shown variable antibody responses to SARS‐CoV‐2 mRNA vaccines, particularly in individuals with ongoing lymphopenia or hypogammaglobulinemia, whereas antigen‐specific T‐cell responses may still be detectable even in the absence of B‐cell recovery. These observations underscore the need to consider both quantitative and qualitative markers of immune recovery when determining the optimal timing for mRNA vaccination after HSCT.

Due to the lack of validated thresholds for lymphocyte subsets, current vaccination guidelines do not recommend timing vaccination based on lymphocyte counts. However, the ECIL guidelines do recommend administering a second dose of influenza vaccine in patients with low lymphocyte counts, without specifying any specific threshold [11, 12].

## 8 Emerging Cellular and Immunotherapies: Implications for Vaccine Responses

Novel immune therapies, such as chimeric antigen receptor (CAR) T cells and bispecific antibodies, also profoundly impact immune reconstitution and vaccination strategies.

CAR T‐cell therapy, targeting CD19 for B‐cell malignancies, induces prolonged B cell aplasia and hypogammaglobulinemia, impairing vaccine responses [75]. Observationnal studies in adults show reduced but measurable antibody response to influenza vaccine, particularly in patients vaccinated prior to CAR‐T cell infusion [76]. Data in pediatric populations remains limited.

Current consensus supports non‐live vaccines from 6 months post‐infusion in patients in remission and off immunosuppressive therapy, while live‐attenuated vaccines are deferred for at least 12 months and only after documented immune recovery without recent intravenous immunoglobulin [77, 78].

Bispecific T‐cell antibodies also induce prolonged B cell depletion, leading to hypogammaglobulinemia and impaired vaccine responses for at least 6 months post treatment [79]. Due to limited pediatric and adults data, vaccination strategies should be personalized [80].

## 9 Discussion

Determining the optimal timing for vaccination after allogeneic HSCT is crucial to reduce vulnerability to infectious complications and to maximize vaccine response. However, immune reconstitution in children after HSCT is highly variable and influenced by multiple factors, including conditioning intensity, stem cell source, GVHD, and age. Current guidelines, largely derived from adult data, do not fully account for this heterogeneity, and evidence in pediatric populations remains limited. Moreover, the most immunologically fragile patients, those with hypogammaglobulinemia and severe GVHD, are often excluded from vaccine studies further restricting the generalizability of available data.

A personalized immune‐guided vaccination approach would ideally optimize protection against VPDs in this vulnerable population. Early pilot studies in pediatric HSCT recipients suggest that strategies tailored to immune recovery can enhance revaccination outcome [70]. Serological monitoring may also help refine vaccine timing and identify children who require additional dose to achieve adequate immunity. Nevertheless, the implementation of individualized schedule is currently constrained by the need for standardized immune‐monitoring tools, additional resources and robust prospective validation.

Until more high‐quality pediatric data become available, adherence to existing post‐HSCT vaccination guidelines remains the safest and most pragmatic approach. When feasible, post‐vaccine serologies, for vaccine with established correlates of protection, can complement guidelines‐based schedules by identifying patients who may benefit from booster doses. Ultimately, well‐designed prospective studies are needed to define immune markers of vaccine responsiveness, determine optimal timing for each vaccine, and evaluate whether personalized schedules can meaningfully improve protection in children after allogeneic HSCT.

## Author Contributions

**Geraldine Blanchard‐Rohner**, **Laure Pittet:** conceptualization. **Hélène Buvelot**, **Geraldine Blanchard‐Rohner:** writing – original draft preparation. **Hélène Buvelot**, **Frederic Baleydier**, **Laure Pittet**, **Geraldine Blanchard‐Rohner:** review and editing. **Hélène Buvelot**, **Frederic Baleydier**, **Laure Pittet**, **Geraldine Blanchard‐Rohner:** validation.

## Funding

The authors have nothing to report.

## Conflicts of Interest

The authors declare no conflicts of interest.

## Data Availability

Data sharing not applicable to this article as no datasets were generated or analysed during the current study.
